# Influence of environmental factors on the spat recruitment of the eastern oyster (*Crassostrea virginica*) along a subtropical river estuary in the Gulf of Mexico

**DOI:** 10.1371/journal.pone.0351746

**Published:** 2026-06-25

**Authors:** Isidro Otoniel Montelongo-Alfaro, Jorge Homero Rodríguez-Castro, Jaime Luis Rábago-Castro, Alberto Carlos Velázquez-Narváez, Filiberto Toledano-Toledano, Roberto Pérez-Castañeda, Alfonso Correa-Sandoval

**Affiliations:** 1 Dirección Académica, Universidad Tecnológica del Mar de Tamaulipas Bicentenario, Soto La Marina, Tamaulipas, México; 2 División de Estudios de Posgrado e Investigación, Instituto Tecnológico de Ciudad Victoria, Tecnológico Nacional de México, Ciudad Victoria, Tamaulipas, México; 3 Facultad de Medicina Veterinaria y Zootecnia, Universidad Autónoma de Tamaulipas, Ciudad Victoria, Tamaulipas, México; 4 Unidad de Investigación en Medicina Basada en Evidencias, Hospital Infantil de México Federico Gómez Instituto Nacional de Salud, Doctores, Cuauhtémoc, Ciudad de México, México; 5 Unidad de Investigación Multidisciplinaria en Salud, Instituto Nacional de Rehabilitación Luis Guillermo Ibarra Ibarra, Ciudad de México, México; 6 Dirección de Investigación y Difusión del Conocimiento, Instituto Nacional de Ciencias e Innovación para la Formación de Comunidad Científica-INDEHUS, Ciudad de México, México; Bigelow Laboratory for Ocean Sciences, UNITED STATES OF AMERICA

## Abstract

Oyster spat recruitment is a key process controlling the persistence of natural populations. However, the relative influence of environmental gradients on spat recruitment remains poorly understood in estuaries of the Gulf of Mexico, especially along the Mexican coast. The aim of this study was to analyze the influence of some environmental factors on the spat recruitment of the eastern oyster (*Crassostrea virginica*) along the subtropical estuary of the Soto la Marina River, Gulf of Mexico. Six sampling sites were selected along the estuary, ranging from 1 km (Site 1) up to 22.8 km (Site 6) from the river mouth. Monthly, samples (April 2020–March 2021) of oyster spat (*C.* *virginica*) were collected from each site using an artificial spat collector. Water temperature, salinity, dissolved oxygen, pH, and Secchi depth were measured at the same time as oyster spat were collected for each site. Spatial and seasonal variations in the number of oyster spat were evaluated using a generalized linear mixed model (GLMM). Additionally, the relationship between spat recruitment and environmental variables was assessed using multiple linear regression analyses. Salinity was the unique environmental variable with significant differences between sites, showing a decreasing gradient from the river mouth to the upstream locations. The mean density of oyster spat decreased significantly from Site 1 to Site 5; however, they were absent at Site 6. Salinity and temperature were the environmental factors that explained the spatio-temporal variation of spat recruitment, showing a positive relationship with spat density on the collectors. Since temperature did not show significant differences between sampling sites, salinity is considered the primary environmental condition influencing the spat recruitment along the estuary. These findings provide a basis for identifying optimal areas and periods for oyster spat collection, supporting efforts in oyster reef restoration and the development of oyster aquaculture.

## Introduction

The eastern oyster (*Crassostrea virginica*) is distributed along the Western Atlantic coast, from the Gulf of St. Lawrence in Canada to the Northern coast of Brazil [1]. The Gulf of Mexico coast accounts for about 70% of Mexico’s annual oyster production, which totals 40,419 tons, mainly from Tamaulipas, Veracruz, and Tabasco. National fisheries statistics indicate that 81% of oyster production in the Gulf is derived from aquaculture practices [2]. This activity relies mainly on enhancing established beds through the addition of oyster shells to increase available settlement substrate, as well as on spat collection, with juveniles subsequently transferred to prepared grow-out areas [3]. The fishery is managed through commercial harvesting permits issued by the federal government (via CONAPESCA). Harvesting typically occurs in shallow lagoonal environments at depths of 0.5–2.5 m, either manually or using wooden oyster tongs fitted with metal rakes. Management measures include a minimum legal size of 70 mm and seasonal closures implemented along the southern Gulf coast [4].

After the reproduction of *C. virginica*, their larvae are part of the zooplankton for two weeks, and they must find a suitable substrate conditions for the settlement during this period; otherwise, they die [5]. Larval settlement at a specific location depends on various factors, such as larval density of the water column, larval transport, currents, and circulation patterns [6]. In turn, these factors, affect the spat recruitment and the extent and location of oyster beds within estuaries [7,8,9].

Therefore, the survival and long-term sustainability of wild oyster populations rely on their reproductive capabilities and the presence of suitable environmental conditions, including available substrate for settlement and recruitment. In certain areas, it has been indicated that the availability of appropriate substrates for settlement, particularly oyster shells, may play a more significant role than larval supply in determining oyster spat recruitment [10,11]. Evidence indicates that *C. virginica* recruitment is greater on natural oyster shell reefs than on alternative substrates [12]. Nevertheless, certain extraordinary environmental events, such as the excessive discharge of freshwater into estuarine ecosystems, can result in the absence of recruitment [13]. On the other hand, the temporal pattern of oyster reproduction directly affects the subsequent recruitment of oyster spat throughout the year [14]. However, temporal variations of the reproduction peaks of oysters appear to be closely related to latitude. For instance, in a study conducted at locations along the north shore of Nova Scotia, Canada, the spawning period for *C. virginica* occurred from June to July [15]. This short spawning period of two months contrasts with the six-month spawning period (May–October) reported for lower latitudes such as coastal Georgia [16]. Conversely, *C. virginica* spawns continuously throughout the year in the tropical regions of the Gulf of Mexico, excluding July and August [17].

Oyster spat can be collected from their natural environment [18] to replenish natural populations or for the production of cultured oysters. However, in both cases, the spat availability is a critical factor. Therefore, knowing the spatial variations of spat abundance in the coastal ecosystem is fundamental in order to identify the best sites for spat collection.

The estuary of the Soto la Marina River, which is located at the central part of the state of Tamaulipas, Mexico, is an important oyster fishing area in this region of the Gulf of Mexico. This coastal ecosystem is a classical positive estuary [19], exhibiting a decreasing salinity gradient from the mouth of the estuary to the upstream zone. In several locations along this ecosystem, oyster beds cover the bottom and are commercially exploited. State-level fishery reports indicate that oyster harvests in Tamaulipas total approximately 364 t per year, valued at approximately 880,000 USD [2]. This estuary contributes roughly 6% of that production [20]; however, it holds potential for increased production through aquaculture, which is currently almost absent in the region. In this context, the availability of oyster spat may be a limiting factor for developing this activity.

The detection of areas with high availability of oyster spat (*C.* *virginica*) and how environmental factors influence their recruitment along Soto la Marina River estuary are ecological aspects that have not been studied. This will aid in improving oyster conservation and management practices, as well as identifying possible spat collecting locations for aquaculture or population restoration. Therefore, the aim of this study was to analyze the influence of several environmental factors on oyster spat recruitment (*C.* *virginica*) along the subtropical estuary of the Soto la Marina River on the coast of the Gulf of Mexico.

## Materials and methods

**Fig 1:**
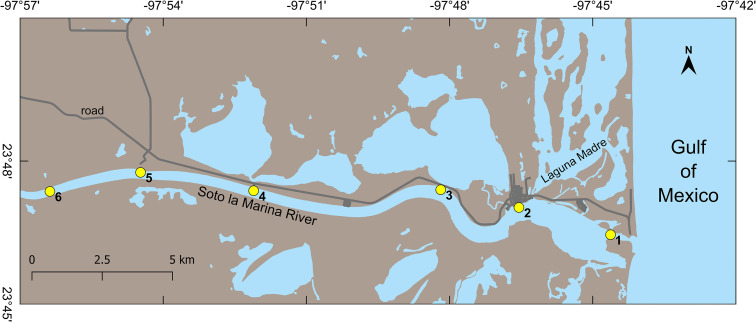
Sampling sites in the estuary of the Soto la Marina River, Gulf of Mexico. Map created by the authors in QGIS (v3.44.8) using publicly available topographic data from INEGI (“Conjunto de datos vectoriales de información topográfica F14B14 La Pesca escala 1:50,000 serie II”; https://www.inegi.org.mx/app/biblioteca/ficha.html?upc=702825224059) and finalized with Inkscape (v1.4.3).

This study was undertaken in the Soto la Marina River estuary (Tamaulipas, Mexico), which is a subtropical coastal ecosystem (23°46’12”–23°47’24” N, 97°44’ 00”–97°56’20” W) that drains into the Gulf of Mexico (Fig 1). The Soto la Marina River flows continuously for about 200 kilometers from the Vicente Guerrero dam to the Gulf.

The study area was delineated based on field observations of oyster shells found in the intertidal zone or on mangrove roots. As a result, six sampling sites were established along the estuary, ranging from 1 km (Site 1) up to 22.8 km (Site 6) from the river mouth. Monthly (from April 2020 to March 2021), samples of oyster spat (*C*. *virginica*) were collected from each sampling site (ca. 2.0 m depth). For each sampling month and site, three horizontal collectors were utilized for the collection of oyster spat under the “Permiso de Acuacultura de Fomento” (Aquaculture Promotion Permit) No. PAF/DGOPA-073/2020 issued by CONAPESCA.

**Fig 2:**
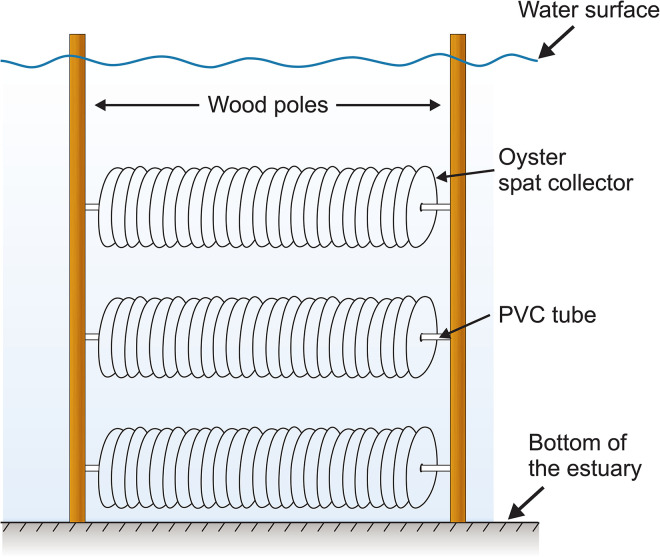
Wooden rack with three oyster spat collectors, installed at each sampling point. Each horizontal spat collector consisted of 25 circular plastic plates mounted along a horizontal PVC tube and positioned at different heights above the substrate (10, 50, and 90 cm).

Each horizontal spat collector consisted of a PVC tube with 25 round plastic plates (’Chinese hat’ type) stacked along the tube. The available area for oyster settlement per each round plate, considering both sides, was 334.6 cm^2^. Therefore, the available area for spat recruitment on each horizontal collector was 0.8365 m^2^, corresponding to the total surface area of the 25 round plastic plates inserted into each PVC tube. Each PVC tube was attached to a pair of wooden poles buried in the substrate to ensure structural stability, forming a rack of three horizontal collectors positioned at different heights above the bottom (10, 50, and 90 cm, respectively) (Fig 2).

The horizontal collectors were dismounted from the rack during each monthly sampling event at each site. The round plates were removed from each horizontal collector for the extraction and counting of spat which consisted of newly settled oysters larger than 2 mm in shell height (distance from the umbo to the opposite margin of the shell) [5]. Therefore, in our study, spat recruitment refers to early juvenile oysters, approximately 2 mm in size and visible to the naked eye, that survived and grew to this size following larval settlement in the collector, which was submerged in the estuary for a month. After the spat were removed, the round plates were washed and placed again in the horizontal collectors to be reinstalled in the rack.

Hydrological variables (water temperature, salinity, dissolved oxygen, and pH) were recorded once a month (at a depth of approximately 20 cm) at each site. These measurements were taken concurrently with the monthly collection of oysters from the collectors. Sampling activities were carried out during the flood tide and high tide. A handheld field instrument (YSI, Pro20) was used to measure temperature (°C) and dissolved oxygen (mg·L^-1^). A refractometer (VEE GEE STX^-3^) and a pH meter (pHep, Hanna) were used to measure salinity (parts per thousand: ppt) and pH, respectively. Secchi depth (cm) readings were also recorded using a Secchi disk (LaMotte 0171). Differences in hydrological variables among sites were assessed using one-way ANOVA or Kruskal-Wallis test, depending on whether the normality assumption (Shapiro-Wilk test) was met. Homogeneity of variances was assessed with Levene’s test. Pairwise comparisons between sites were performed using Tukey’s HSD test (Tukey-Kramer adjustment for unequal sample sizes) when significant differences were detected with ANOVA.

To examine variation in the number of oyster spat collected across different sampling sites and seasons, a generalized linear mixed model (GLMM) with a negative binomial distribution and a logarithmic link function was employed. We initially fitted a Poisson GLMM, but due to high overdispersion and elevated AIC/BIC values, it was replaced with a negative binomial model to account for extra-Poisson variability (θ). The analysis was conducted using the glmmTMB function from the *glmmTMB* package [21] in R software [22].

For this model, we used spat count data from each horizontal collector deployed at Sites 1–5. Site 6 was excluded from the analysis because recruitment was consistently absent at this location, and the analysis focused on comparing sites with active recruitment. The dependent variable was the number of oyster spat (SpatN) collected per each individual horizontal collector. The fixed effects included the factors Site (five levels, corresponding to specific locations within the estuary) and Season (four levels: spring, summer, autumn, and winter). To account for additional sources of variability, random effects were included for Month (the calendar month in which sampling was conducted), and Depth (the depth at which each horizontal collector was positioned).

The adjusted model was as follows:

SpatN ~ Site + Season + (1 | Month) + (1 | Depth)

The coefficients of the fixed effects were evaluated using Wald tests (z-tests), and the overall significance of each fixed factor (Site and Season) was assessed using type III Wald chi-square tests [23]. Post hoc pairwise comparisons between levels of fixed factors were conducted using Tukey-adjusted contrasts via the *emmeans* package [24]. Overdispersion, residual diagnostics, and model fit were also assessed.

We analyzed the relationship between mean spat abundance at each site (averaged across the three horizontal collectors) and the distance upstream from the river mouth using a nonlinear model of the form $Y=ae^{\left(-bX\right)}$. The model was fitted in R using nonlinear least squares (Gauss–Newton algorithm) and the significance of the estimated parameters was determined. Additionally, 95% confidence intervals were calculated using the *confint* function with the profile likelihood method [22].

A multiple regression analysis was performed to determine those predictor variables (environmental variables: temperature, dissolved oxygen, salinity, pH, and Secchi depth) affecting the spat recruitment to the estuary. The above analysis was conducted using monthly data from all sampling sites (n = 63 observations), based on spat abundance, derived from the average of three horizontal collectors deployed at each site. Data were log-transformed using log(x + 1) prior to analysis. Environmental variables were lagged by one month relative to spat abundance to relate spat recruitment to environmental conditions from the preceding month, which approximately corresponds to the period of larval settlement. We assumed that the spat collected in our study were approximately one month old at most, based on their size, which ranged up to 10–12 mm in shell length [25].

Multiple linear regression models were compared, including a complete model and a simplified version containing only significant predictors. Model fit was evaluated using AIC, BIC, and ANOVA for nested models to determine whether the inclusion of additional predictors resulted in a significant improvement. To assess the potential non-additive effects, interactions among the predictor variables were also tested. As none were statistically significant (p > 0.05), only the main effects were included in the final model. Assumptions of low multicollinearity among predictor variables and normally distributed residuals were assessed and met (Variance Inflation Factors ≤ 1.7; Shapiro–Wilk test: p > 0.05). We used the *visreg* package to generate partial regression plots for the significant predictors, allowing visualization of their individual effects on the response variable while controlling for the influence of other variables. All statistical analyses described above were conducted using R software [22].

## Results

### Environmental factors

Salinity exhibited a sharply decreasing gradient from the river mouth to the upstream locations; its mean value (± SE) was significantly highest at Site 1 (30.5 ± 1.7 ppt) and lowest at Site 6 (6.4 ± 1.8 ppt). Along the estuary, the mean temperature ranged from 23.3 to 26.9°C, dissolved oxygen from 6.6 to 7.3 mg·L^-1^, pH from 7.6 to 7.9, and Secchi depth from 39.8 to 58.0 cm; however, none of these variables differed significantly among sites (Table 1). Monthly values of the environmental variables for each site are provided as supplementary material (S1 Fig).

**Table 1:** Spatial Variation (Mean ± SE) in Temperature, Salinity, Dissolved Oxygen, pH and Secchi Depth at Six Sampling Sites Along the Soto la Marina River Estuary (Tamaulipas, Mexico). The Data Were Collected During 12 Monthly Samplings at Each Site (n = 12 per site).

Environmental factor	Site						Test statistic	*p* value
	1	2	3	4	5	6		
Temperature (°C)	24.4 ± 1.4	23.3 ± 1.4	25.8 ± 1.3	26.5 ± 1.2	26.8 ± 1.3	26.9 ± 1.3	H(5) = 2.85	0.723
Salinity (ppt)	30.5 ± 1.7 ^a^	21.8 ± 2.5 ^ab^	16.8 ± 2.7 ^bc^	9.8 ± 2.4 ^cd^	8.1 ± 2.3 ^cd^	6.4 ± 1.8 ^d^	F(5, 66) = 17.02	< 0.001
Dissolved oxygen (mg·L ^− 1^)	7.3 ± 0.5	7.3 ± 0.3	7.0 ± 0.3	6.7 ± 0.2	6.6 ± 0.4	6.9 ± 0.3	H(5) = 4.19	0.523
pH	7.6 ± 0.2	7.8 ± 0.2	7.7 ± 0.2	7.9 ± 0.2	7.9 ± 0.2	7.9 ± 0.2	H(5) = 5.33	0.377
Secchi depth (cm)	54.6 ± 7.5	46.8 ± 4.5	58.0 ± 4.0	48.0 ± 5.9	49.5 ± 4.5	39.8 ± 4.7	F(5, 66) = 1.43	0.224

Notes: p-values for Kruskal–Wallis (temperature, dissolved oxygen, pH) and ANOVA (salinity, Secchi depth) are reported.

### Oyster spat recruitment

**Fig 3:**
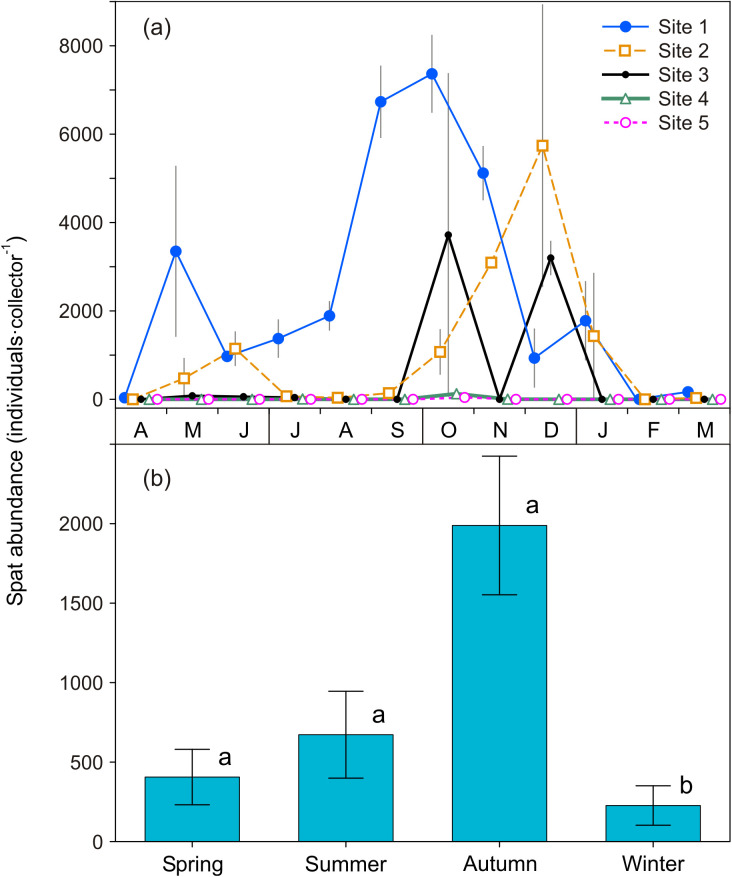
Monthly (a) and seasonal (b) variations in the number (mean ± SE) of oyster spat observed on collectors. Descriptive values are shown for illustration, while statistical comparisons among seasons were conducted using a generalized linear mixed model (GLMM). Significant differences were detected among seasons (Type III Wald chi-square test: χ² = 19.35, df = 3, *p* < 0.001). Results of pairwise contrasts of estimated marginal means (emmeans) are also shown, with different letters indicating seasons that differed significantly (p < 0.05) from each other.

Oyster spat were continuously recruited to the artificial collectors throughout the year at one of the sites (with the exception of February 2021), with significant seasonal differences detected (GLMM, Type III Wald χ² = 19.35, df = 3, p < 0.001). Although the highest recruitment peaks were observed from September to December (summer and autumn), oyster spat abundance did not differ significantly from spring to autumn. However, recruitment decreased significantly in winter (Tukey-adjusted pairwise comparisons, p < 0.05; Fig 3).

**Fig 4:**
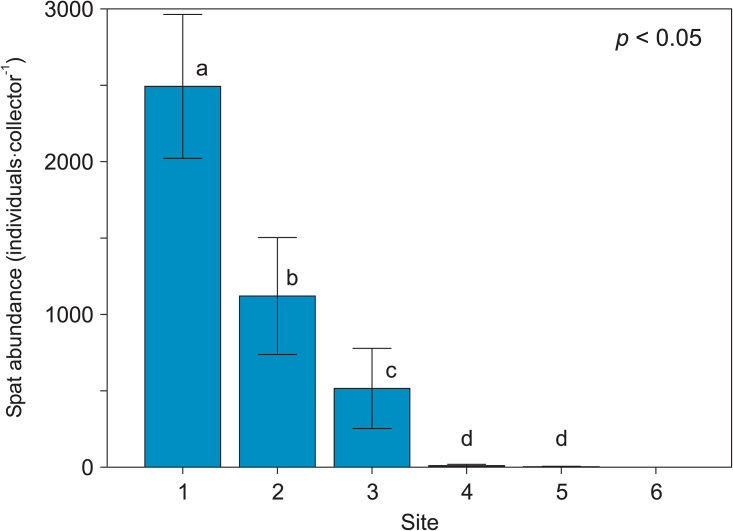
Observed spatial variation in the abundance (mean ± SE) of oyster (*Crassostrea virginica*) spat along sites in the Soto la Marina River estuary. Descriptive values are shown for illustration, while significant differences among sites were assessed using a generalized linear mixed model (GLMM). Significant differences were detected among sites (χ² = 120.12, df = 4, p < 0.001). Results of pairwise contrasts of estimated marginal means (emmeans) are also shown, with different letters indicating sites that differed significantly (p < 0.05) from each.

**Fig 5:**
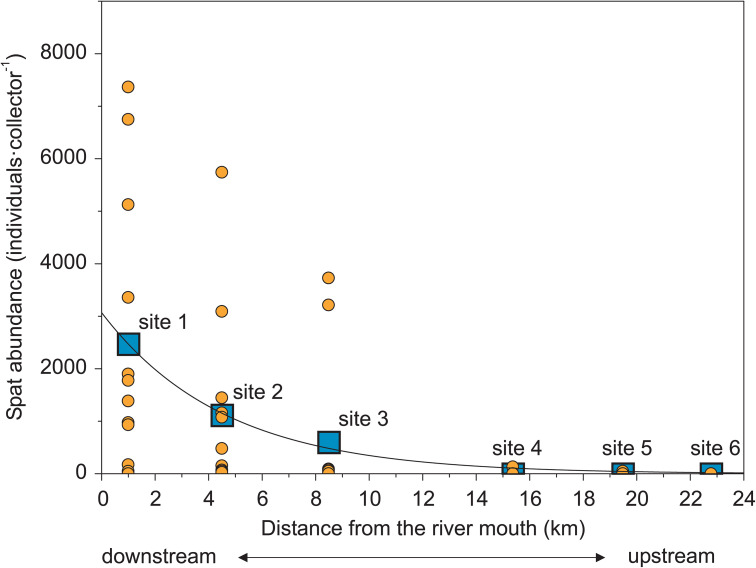
Abundance of oyster spat (mean of three horizontal collectors per site) as a function of distance from the river mouth. An exponential decay function (*Y* = *a e*^-*bx*^) was fitted to the data. Both model parameters were significant. Data markers represent abundance values for each site (squares, overall mean abundance; circles: monthly abundance). The estimated model parameters *a* and *b* were 3069.95 (95% CI: 1993.15 to 4520.46) and −0.22 (95% CI: −0.46 to −0.11), respectively.

The mean abundance (± SE) of oyster spat settled on the collectors declined markedly from Site 1 (2493 ± 470.8 individuals·collector^-1^) to Site 5 (3.4 ± 2.0 individuals·collector^-1^). Spat were absent at Site 6 during the study period and not considered in the analysis. Variation in spat recruitment among Sites 1–5 was significant (GLMM, Type III Wald χ² = 120.12, df = 4, p < 0.001), with recruitment significantly higher at Site 1 and lower at Sites 4 and 5 (Tukey-adjusted pairwise comparisons, p < 0.05; Fig 4). Indeed, recruitment showed a pronounced exponential decline along the estuary, with values progressively decreasing upstream as the distance from the mouth increased (Fig 5).

### Relationship between environmental factors and oyster spat recruitment

**Fig 6:**
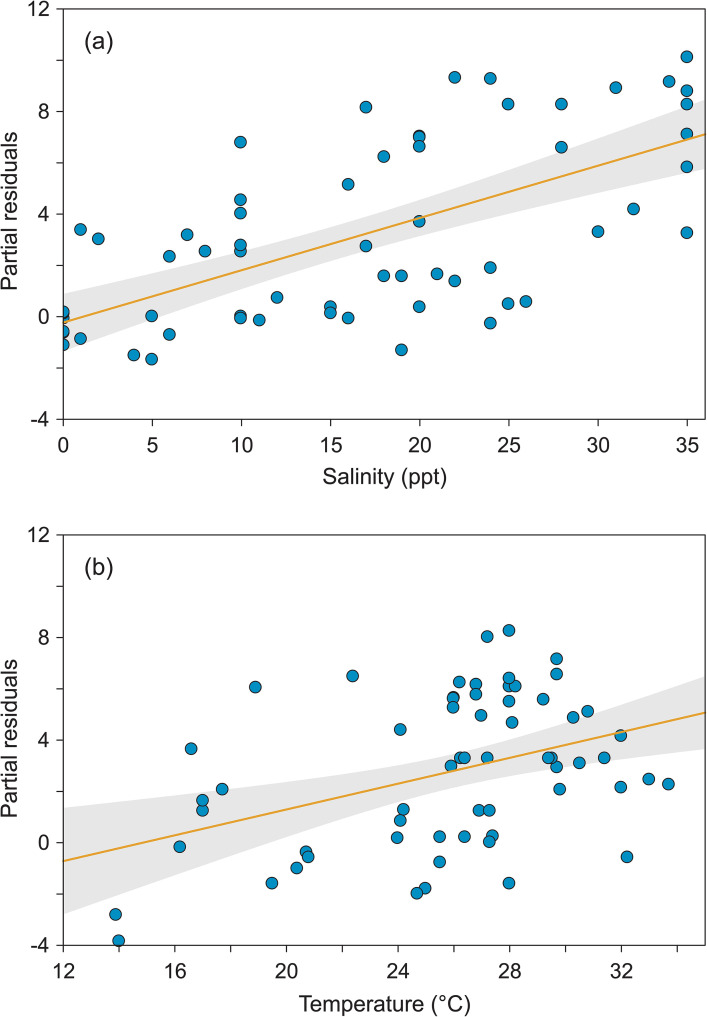
Partial relationships between oyster spat abundance and (a) salinity and (b) temperature (both variables lagged by one month) in the Soto la Marina River estuary, based on the reduced multiple linear regression model. The Y-axis shows the partial residuals of log(spat per collector + 1). Continuous lines show the predicted values from the model, with gray bands representing 95% confidence intervals.

Salinity and temperature were positively related to the number of oyster spat collected in the estuary, according to the multiple linear regression analysis, which utilized one-month-lagged data for all environmental variables. In the full model, salinity showed a significant positive effect (p < 0.001), whereas temperature exhibited a marginally non-significant positive effect (p = 0.056). None of the remaining variables (oxygen, pH, Secchi depth) were significant predictors. In the reduced model, which included only salinity and temperature, both variables were significant positive predictors of spat abundance (p < 0.01), accounting for 37.9% of the variance (Table 2). Partial regression plots (Fig 6) further illustrated the relationships between spat abundance and each predictor while controlling for the other variable in the model, showing the direction and relative strength of the effects along with 95% confidence intervals.

**Table 2:** Regression Coefficients (± SE), 95% Confidence Intervals, and Model Fit Statistics for Full and Reduced Multiple-Regression Models Relating One-Month-Lagged Environmental Predictors to *Crassostrea virginica* Spat Recruitment (Individuals·Collector^-1^) Along the Soto la Marina River Estuary.

Full model				
	Predictor	Estimate	95% CI	p-value
	(Intercept)	7.739	18.114	0.396
	Temperature (°C)	0.178	0.182	0.056
	Oxygen (mg·L ^− 1^)	−0.256	0.600	0.397
	pH	−1.357	1.801	0.137
	Secchi depth (cm)	−0.018	0.037	0.348
	Salinity (ppt)	0.215	0.069	< 0.001
	Adjusted R^2^ = 0.386; F_5,59_ = 9.06; p < 0.001			
Reduced model				
	Predictor	Estimate	95% CI	p-value
	(Intercept)	−7.037	4.611	0.003
	Temperature (°C)	0.252	0.156	0.002
	Salinity (ppt)	0.204	0.064	< 0.001
	Adjusted R^2^ = 0.379; F_2,62_ = 20.53; p < 0.001			

## Discussion

### Oyster biology and reproductive patterns

The temporal pattern of spat density observed in the present study coincided with the main spawning periods of *Crassostrea virginica* reported for other estuarine ecosystems in the Gulf of Mexico, including tropical systems such as Tamiahua and Mecoacan lagoons (Mexico) [17,26] and subtropical systems such as Caloosahatchee estuary (South Florida, USA) [27]. Although temporal variability in spawning activity was not directly assessed in this study, previous research in these regions suggests a close association between seasonal peaks in spat recruitment and reproductive cycles. Consequently, the observed temporal pattern of spat recruitment in the Soto la Marina estuary may be associated with seasonal variation in oyster spawning, as documented in other Gulf of Mexico ecosystems.

### Spatial patterns of spat settlement

Concerning the spatial variations of oyster spat recruited to the collectors, a decreasing pattern of spat per collector was observed from the mouth to the upstream sites, i.e., as distance from the river mouth increased, spat density decreased. The decreasing salinity gradient from the estuary mouth (Site 1: 30.5 ± 1.75 ppt) to upstream (Site 6: 6.4 ± 1.83 ppt) could partially explain this spatial pattern in spat density.

Similarly, in the Caloosahatchee estuary, the spat recruitment of *C. virginica* increased at sites located downstream [27,28]. Likewise, in Chincoteague Bay (Mid-Atlantic coast, USA), oyster larval settlement was much higher at sites located closest to the inlets, i.e., those sites with a greater marine influence [29]. In both above cases, and as observed in our study, the highest spat recruitment occurred where salinity was highest. Conversely, oyster spat settlement was lower at low-salinity sites in the St. Lucie Estuary (Florida) compared to an adjacent high-salinity coastal ecosystem [30]. Spat settlement and freshwater discharge dynamics are closely related because freshwater flow modifies salinity, as documented in the Mobile Bay-eastern Mississippi Sound system [31].

Although adult oysters might be found in the upstream zone beyond Site 3, the low salinity in that area (from 6.4 to 9.8 ppt, Table 1) could explain the low density or absence of spat recruitment from Sites 4–6. The higher spat recruitment found at downstream sites in an estuary could be due to the number of spawning oysters and the flushing of oyster larvae to downstream locations due to currents in the downstream direction [27].

Regardless of potential larval dispersal, sites near natural oyster reefs, where spawning adults are located, tend to have higher spat densities [32]. Live and dead shells on and near reefs offer suitable substrate for larval settlement. Indeed, significant relationships between spat recruitment and the area of substrate available for larval settlement have been documented, especially in high-salinity areas [10]. Oyster recruitment is usually influenced by the substrate availability and reef surface area. This is because larvae generally require hard substrate, preferably oyster shells, for larval settlement. Several studies have documented positive relationships between reef surface area, shell availability, and spat recruitment, suggesting that substrate limitation may play an important role in regulating oyster population dynamics [7,10,33]. According to the above, an increase in adult abundance may increase shell accumulation, which in turn could enhance larval settlement and subsequent reef accretion [5,11].

The Soto la Marina River has a continuous flow of freshwater, which increases during the rainy period from late summer to early autumn [34]. The period of increased water flow in the downstream direction coincides with the peak reproductive activity reported for this oyster species [17,26]. This suggests that the river flow might contribute to the movement of larvae downstream, affecting the pattern of oyster spat recruitment along the estuary.

### Environmental variability and implications for oyster ecology

Salinity was the only environmental variable that showed significant differences among the study sites. The marked salinity gradient along the estuary is a consequence of its hydrodynamics, where riverine freshwater mixes with seawater entering from the Gulf of Mexico due to tidal influence. In fact, according to its hydrological characteristics, the Soto la Marina River estuary is considered a positive estuary because freshwater inflow from the river, groundwater, and rainfall exceed evaporation [35]. Therefore, a decreasing salinity gradient from the mouth to upstream areas is common in this type of estuary [36].

Salinity is widely recognized as a primary factor regulating the distribution, survival, growth, and reproduction of oysters, with physiological optima reported to range between 12 and 24 ppt [37]. According to experimental studies, it has been observed that low or fluctuating salinities can reduce early juvenile oyster growth [38]. Likewise, observational studies have reported a reduction in oyster shell and muscle growth in estuarine environments with low salinity [39]. Sites with intermediate salinities (approximately 17–22 ppt) in the Soto la Marina River estuary fall within the ranges associated with high growth efficiency and favorable reproductive conditions for *C. virginica* [37,40].

Low salinity conditions represent a critical osmotic stressor for *C. virginica* oysters, strongly affecting their different life stages. Experimental studies have shown that salinities below 10 ppt completely inhibit hatching rate and reduce larval survival and settlement, as well as the survival and growth of spat [41,42]. In adult oysters, low salinity conditions induce prolonged valve closure, resulting in reduced feeding activity [43], and when exposure persists for several weeks, it may result in massive mortality [44]. In our study, the extremely low salinity conditions recorded at the upstream sites, especially at Site 6 (mean: 6.4 ppt), likely exceeded the tolerance range required for oyster recruitment and survival. This could explain the absence of spat at this site. Therefore, the marked upstream decline in spat abundance may reflect not only spatial variation in reproductive suitability, but also physiological restrictions imposed by persistent low-salinity conditions on larval and early post-settlement survival.

In the Hudson River estuary (New York, USA), it has been reported that *C.* *virginica* oysters usually show higher reproductive activity in sites with salinities between 20 and 30 ppt [45], similar to salinities recorded at sites 1 and 2 in this study. The recruitment peaks observed in the present study were mainly recorded at sites with salinities of approximately 22 ppt or higher. Although higher spat recruitment could be related to reproductive activity, larval transport processes associated with freshwater discharge and tidal cycles [46,47] may also play an important role in concentrating larvae toward downstream, higher-salinity areas. This larval transport mechanism is consistent with the river-dominated nature of the estuary under study. In addition, it has been reported that oyster spawning decreases where salinity is < 15 ppt [45]. Nevertheless, the lack of information on larval connectivity and oyster metapopulation dynamics within the estuary constrains our ability to fully discern the relative contribution of local reproductive output compared with physical larval transport. To date, no studies have evaluated oyster metapopulation dynamics within the Soto la Marina River estuary. This underscores the need for future research to explore larval dispersal and connectivity patterns in this system.

Low salinity is also associated with reduced predation pressure and lower disease prevalence, favoring population performance even though individual growth rates are not maximized [40,48]. Sites located downstream, with salinity close to that of the sea (ca. 35 ppt), can generate rapid growth but are often associated with higher risks of predation and pathogen infection. Conversely, upstream sites with salinities below 10 ppt may reduce predation but also cause greater physiological stress and slower growth due to higher energetic demands of osmoregulation [37,40,48].

Water temperature varied moderately along the estuary (23.3–26.9 °C), falling within ranges considered optimal for the metabolic activity, filtration, and growth of *C. virginica*. This indicates that, during the sampling period, temperature was not a limiting factor for oyster performance in the study area [37].

According to the multiple linear regression analysis, salinity and temperature were the main environmental variables that explained spat abundance in the estuary. Temperature may be related to reproductive activity throughout the year [26,49], but since it did not differ among sites, salinity was considered the primary environmental condition influencing the abundance of spat recruitment throughout the estuary. Although salinity and temperature were identified as the main environmental variables associated with spat recruitment in this study, oyster recruitment is a complex process influenced by multiple interacting factors. Additional important variables not included in our study were food quantity and size, substrate availability, distribution and biomass of adult oyster stocks, reef surface area, local hydrodynamics, and larval transport processes [47]. Previous studies have indicated that food availability can influence larval development, while substrate limitation and reef structural complexity can strongly affect larval settlement success [10,50–52], while adult population size and reef area may play key roles in larval supply and stock–recruitment dynamics [53]. In addition, local circulation patterns and estuarine hydrodynamics can regulate larval retention, dispersal, and connectivity among reef areas [46,47,54]. These factors likely interact with salinity and temperature to shape recruitment variability within the estuary. Therefore, future studies that incorporate environmental variability, reef structure, oyster population attributes, and hydrodynamic processes would improve our understanding of spat recruitment dynamics in the Soto la Marina River estuary.

Dissolved oxygen concentrations remained high and stable (>6.5 mg L ⁻ ¹), falling within ranges that are not considered limiting to oyster physiology [37]. For oyster reefs, lower mortality has been documented in locations where oxygen concentrations exceed 3.9 mg L ⁻ ¹ [55]. pH values also remained stable (≈7.6–7.9) throughout our study, consistent with conditions suitable for calcification and growth of *C. virginica* [5,37].

Other studies in the Gulf of Mexico have reported reduced *C. virginica* recruitment at salinities below 10 ppt and increased recruitment when salinity exceeds 10 ppt, particularly at temperatures above 25 °C [56]. Similarly, positive correlations between salinity and *C. virginica* spat density have been documented in a Texas estuary [57] as well as in Chincoteague Bay, where larval settlement of *C.* *virginica* was highest at salinities >30 ppt [29]. In a similar manner, a positive relationship between salinity and spat density of *Crassostrea* oysters has been documented in other parts of the world [58]. In contrast to Mid-Atlantic coastal bays, where pH and DO have been linked to reduced oyster larval recruitment [29], pH and DO in the Soto la Marina River estuary exhibited narrower ranges and were not significant predictors of recruitment. This difference may reflect both the limited variability observed in our study area and underlying morphogeographic differences between mid-Atlantic coastal bays and the river-dominated estuary.

The spatial and temporal patterns in oyster spat abundance may also be influenced by the condition and extent of oyster reefs throughout the estuary. However, information on reef distribution and surface area is currently unavailable for the Soto la Marina River estuary. Assessment of these reef attributes would help clarify their contribution to spat recruitment dynamics.

Although clear spatial and temporal patterns of spat recruitment were observed in the Soto la Marina River estuary, our results should be interpreted within the context of a single year of sampling conducted in this estuarine system. Salinity conditions in the estuary can vary substantially from year to year due to differences in precipitation, freshwater discharge, and broader climatic variability, including El Niño Southern Oscillation events, all of which may influence estuarine hydrodynamics and oyster recruitment dynamics [59,60]. For example, it has been documented that interannual changes in freshwater inflows in the Yangtze River estuary modified salinity regimes, ultimately affecting oyster population dynamics and reef-associated communities [61]. Therefore, the salinity–recruitment relationships documented in our study area should be viewed as representative of the environmental conditions observed during the study period. Long-term monitoring across multiple years would help determine the interannual consistency of spat recruitment dynamics within the estuary.

### Implications for fisheries, aquaculture, and restoration

The environmental patterns identified in this study may provide practical guidance for oyster management, aquaculture, and restoration projects in the Soto la Marina River estuary. The consistently high spat recruitment observed at downstream sites, where salinity ranged from approximately 17–30 ppt, suggests that these areas may be the most suitable locations for spat collection and the establishment of restoration reefs. These downstream areas likely serve as the primary natural sources of oyster seed within the estuary, making them particularly important for fisheries, aquaculture, and restoration initiatives. This finding is consistent with previous research highlighting the importance of environmental suitability and site selection for restoration success [52,62]. Conversely, upstream areas (Sites 4–6), characterized by low salinity conditions and negligible spat recruitment, are unlikely to support successful natural recruitment and are therefore less suitable for reef construction or spat collection programs.

Additionally, seasonal patterns in spat recruitment suggest that deploying artificial collectors during late summer and autumn in downstream zones near the estuary mouth could maximize spat availability for aquaculture or restoration purposes. Identifying when and where recruitment peaks occur may help optimize spat collection efforts and reduce pressure on natural reefs.

Our findings can help local fisheries managers and oyster producers identify spatiotemporal windows with greater potential for sustainable oyster production and restoration under changing estuarine conditions in this subtropical estuary of the Gulf of Mexico. Furthermore, maintaining stable salinity conditions in downstream areas, particularly by avoiding prolonged low-salinity events during the reproductive season, may be essential for sustaining natural recruitment.

## Conclusions

The estuary exhibited clear spatial and temporal patterns of *Crassostrea virginica* spat recruitment along the Soto la Marina River estuary. Spat abundance was consistently greater at downstream sites with higher salinity (≥ 22 ppt, near the river mouth), showing seasonal variability, with a peak recruitment occurring in autumn. These results emphasize the importance of downstream estuarine areas as key zones for oyster settlement and early survival in this river-dominated estuary. Among the environmental variables evaluated, salinity was identified as the primary driver of recruitment, with temperature playing a secondary role, whereas pH and dissolved oxygen showed limited variability and no detectable influence.

The spat recruitment pattern observed in this study demonstrates the value of estuarine salinity gradients in structuring oyster recruitment. These findings provide practical guidance for oyster aquaculture and restoration by identifying periods and locations with higher availability of oyster seed in the estuary. In particular, downstream areas near the estuary mouth may represent priority zones for spat collection, reef restoration, and sustainable oyster production.

## Supporting information

S1 Fig Temporal variation of environmental variables at six sampling sites along the Soto la Marina River estuary (Mexico) from April 2020 to March 2021. Panels show temporal changes per site in water temperature (°C), salinity (ppt), dissolved oxygen (mg·L^-1^), pH, and Secchi depth (cm).
(DOCX)

S1 Data Minimal dataset of environmental and spat recruitment variables. This Excel workbook contains the underlying data used in the study, organized across five spreadsheets: 1) Environmental data; 2) Spat count for GLMM; 3) Spat abundance vs Distance; 4) Spat vs Enviro data for MLR; and 5) Partial residuals data.
(XLSX)
