# Supplementary material for: Influence of environmental factors on the spat recruitment of the eastern oyster (Crassostrea virginica) along a subtropical river estuary in the Gulf of Mexico
Source: PLoS One. 2026 Jun 25;21(6):e0351746. doi: 10.1371/journal.pone.0351746 (PMC13298764; doi:10.1371/journal.pone.0351746)

**S1 Fig**. Temporal variation of environmental variables at six sampling sites along the Soto la Marina River estuary (Mexico) from April 2020 to March 2021. Panels show temporal changes per site in water temperature (°C), salinity (ppt), dissolved oxygen (mg·L^-1^), pH, and Secchi depth (cm).


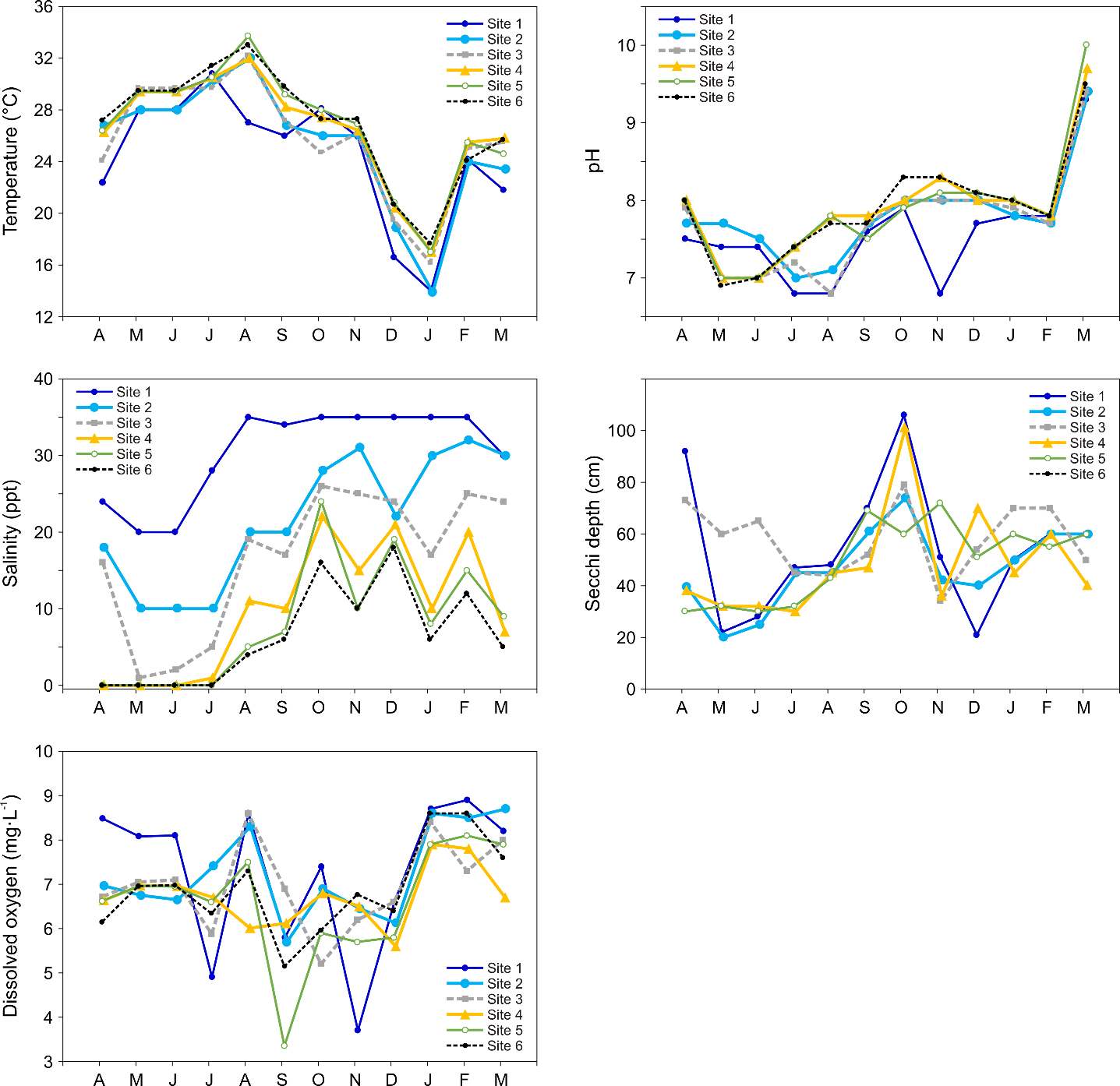

Supplement: S1 Fig — Panels show temporal changes per site in water temperature (°C), salinity (ppt), dissolved oxygen (mg·L-1), pH, and Secchi depth (cm). (DOCX) [file pone.0351746.s001.docx]
